# The Dusk Chorus from an Owl Perspective: Eagle Owls Vocalize When Their White Throat Badge Contrasts Most

**DOI:** 10.1371/journal.pone.0004960

**Published:** 2009-04-08

**Authors:** Vincenzo Penteriani, Maria del Mar Delgado

**Affiliations:** 1 Department of Conservation Biology, Estación Biológica de Doñana, Seville, Spain; 2 Laboratory of Ecological and Evolutionary Dynamics, Department of Biological and Environmental Sciences, University of Helsinki, Helsinki, Finland; University of Lethbridge, Canada

## Abstract

**Background:**

An impressive number of studies have investigated bird vocal displays, and many of them have tried to explain the widespread phenomenon of the so-called dawn and dusk chorus, the sunrise and sunset peaks in bird song output. As many as twelve non-exclusive hypotheses have been proposed to explain why twilight peaks in vocal display might be advantageous; but, even after more than two decades of study, the basis underlying the dusk and dawn chorus is still unclear. Moreover, to date, the majority of studies on this topic have focused on songbirds.

**Methodology/Principal Findings:**

We investigate here a novel hypothesis on why nocturnal birds with patches of white feathers call at twilight. We propose that white plumage patches and the timing of visual signaling have co-evolved to maximize the effectiveness of social communication such as the dusk chorus. This hypothesis centers on the recent discovery that eagle owls can adopt specific forms of visual signaling and is supported by the observation that adult eagle owls possess a white throat badge that is only visible during vocal displays. By monitoring the calling of eagle owls at dusk, a peak time for bird call output, we found that white throat badges contrasted most with the surrounding background during the owls' twilight chorusing.

**Conclusions/Significance:**

Crepuscular and nocturnal species appear to have evolved white patches that, shown in association with vocal displays, allow them to communicate in dark surroundings. The evolution of a white badge that operates jointly with call displays at dawn and dusk may be relevant to the eagle owls' social dynamics. Our explanation for the dusk chorus may possibly represent an overlooked but common pattern of signaling among crepuscular and nocturnal birds that combine patches of white feathers with twilight displays. Furthermore, our findings could be relevant to songbirds that breed in dark forest habitats and have contrasting white badges, as well as birds living in open habitats and showing contrasting bars.

## Introduction

One of the most investigated topics in behavioral ecology is that of birds and their vocal behavior in general, and songbirds and their songs in particular. Numerous studies have sought to explain the amazingly widespread phenomenon of the so-called dawn and dusk chorus [1], [2], i.e. the sunrise and sunset peaks in bird song output. As many as twelve non-exclusive hypotheses have been proposed to explain why twilight peaks in vocal display might be advantageous [1], [2], [3]. However, even after more than two decades of study, the basis underlying the dusk and dawn chorus is still unclear, in part because the complexity of this behavioral pattern seems to reflect different but non-conflicting needs within the same species [2], [4]. To date, the majority of studies on this topic have focused on songbirds, which might contribute to a biased view of bird vocal communication. It seems likely that if multiple hypotheses are necessary to explain songbird dawn and dusk choruses, many others could be necessary to explain the functions of vocal signaling at sunrise and twilight in other bird families. Until we gain a better understanding of daily patterns in signaling by as many as possible bird families, we will not completely understand why birds sing. In addition, we need to examine nonvocal means of communication and how they might be associated with vocalization. It is well-known that diurnal birds use an astonishing variety of color signals to visually communicate: did crepuscular and nocturnal species find a way to communicate by visual signals? Our present study shows that crepuscular and nocturnal species appear to have evolved white patches that, shown in association with vocal displays, allow them to communicate in dark surroundings.

Previous empirical studies have shown that ambient light plays a role in the evolution of color patterns and signals [5], [6], [7]. Therefore, any visual signal used around dawn and dusk should optimize the use of the available light. In this context, achromatic plumage patches are the best candidates for crepuscular signaling, as contrast is more important than color. In fact, the setting or rising sun forms the best light angle for using a white patch as a high-contrast signal against a dark background [5], [8], [9]. Not surprisingly, brightness contrast is a very common animal strategy for enhancing conspicuousness [9].

By studying the nocturnal eagle owl *Bubo bubo* that has been shown to perform dawn and dusk chorusing behavior [10], [11], [12], we herein investigate a novel hypothesis on why nocturnal birds with patches of white feathers call at twilight. We propose that white plumage patches and the timing of visual signaling have co-evolved to maximize the effectiveness of social communication such as the dusk chorus. This hypothesis centers on the recent discovery that eagle owls can adopt specific forms of visual signaling [13], [14], [15], and is supported by the observation that adult eagle owls possess a white throat badge that is only visible during vocal displays, when the throat is repeatedly inflated and deflated (e.g. coverable badges [16]; Video S1).

## Results

The white throat badge contrasted most with the surrounding background and body during the owls' twilight chorusing (Figure S1). The levels of brightness contrast of white badge *vs*. background averaged 117.1±3.0, 138.8±3.0 and 37.7±4.4 during the pre-calling, calling and post-calling periods, respectively (F_2,482_ = 276.7, P<0.0001; Table 1). Similarly, the contrast between the badge and the body was significant between the calling and post-calling periods, but not between the pre-calling and calling periods (F_2,482_ = 291.7, P<0.0001;Table 1): the levels of brightness contrast of white badge *vs*. body averaged 139.6±6.0, 128.8±2.5 and 36.7±4.3 during the pre-calling, calling and post-calling periods, respectively. These two models explained 74.1% and 73.4% of the original deviance, respectively.

**Table 1:** The main features of the two GLIMMIXs testing for the differences of the white badge versus background and white badge versus owl body during the pre-calling period, when owls were calling and during the post-calling period (see text for additional information).

Parameters	Parameter estimate±SE	*P*	% deviance explained
**Model: white badge ** ***vs*** **. background**			**74.1**
Pre-calling period a	116.8±5.4	0.0001	
Calling period a	126.6±5.5	0.0001	
Post-calling period a	37.7±5.7	0.0001	
Male owls b	559.1±183.8	0.001	
Intercept	37.7±5.7		
**Post-hoc tests**			
Pre-calling *vs*. calling periods		0.05	
Pre-calling *vs*. post-calling periods		0.0001	
Calling *vs*. post-calling periods		0.0001	
**Model: white badge ** ***vs*** **. body**			**73.4**
Pre-calling period a	117.7±4.6	0.0001	
Calling period a	118.5±4.7	0.0001	
Post-calling period a	36.7±4.9	0.0001	
Male owls b	376.2±124.1	0.0001	
Intercept	36.7±4.9		
**Post-hoc tests**			
pre-calling *vs*. calling periods		0.9	
pre-calling *vs*. post-calling periods		0.0001	
calling *vs*. post-calling periods		0.0001	

a fixed effects, categorical variable

b random effect

Differences among periods are shown as least square means±S.E. from the mixed models (LSMEANS statement in SAS).

## Discussion

As predicted, eagle owls perform their dusk vocal displays under the light conditions that best allow them to visually communicate with conspecifics through their badges. Due to incomplete knowledge of the physiological functioning of eagle owls, there are several different proximate mechanisms that can also contribute to the time of calling (e.g. peaks in testosterone before sunrise), but most of the main hypotheses previously used to explain the dawn and dusk chorus in birds [1] do not seem sufficient to fully explain the twilight behavior of eagle owls, at least when employed without the support of a visual signaling hypothesis. In fact, because this owl performs dawn and dusk choruses throughout the entire year, it seems difficult to believe that such calling solely represents a method of regulating daily hormone levels depending on the immediate social situation (i.e. self-stimulation hypothesis), attracting females (i.e. mate-attraction hypothesis), stimulating reproductive development (i.e. mate-stimulation hypothesis), or guarding mates (i.e. mate-guarding hypothesis). These hypotheses would seem unlikely because the mate fidelity of territory owners is generally expected to be stronger in eagle owls than in songbirds, and mate attraction, stimulation or guarding during or after the dawn chorus is nonsensical because after sunrise individuals retreat to their diurnal roosts to rest. Finally, since the eagle owl is a top nocturnal predator, it does not have to call under low light conditions to better avoid predation or because foraging is limited and low light levels could interfere with the ability to search for prey (as appears to be the case for Passerines; *low predation* and *inefficient foraging hypotheses*, respectively). We could further discard the inefficient foraging hypothesis because the activity peaks of the rabbit *Oryctolagus cuniculus* [17], the main prey of the eagle owl in Mediterranean regions [18], [19], partially overlaps with the eagle owls' dawn and dusk choruses.

Therefore, our findings concerning the patterns of vocal signaling allows us to hypothesize that eagle owls may principally vocalize at twilight because this timing of signaling maximizes the visual contrast of the white feathers associated with their vocal displays. Consequently, visual signaling may represent an important aspect of conspecifics communication. Under such a viewpoint, the evolution of a white badge that operates jointly with call displays at dawn and dusk may be relevant to the eagle owls' social dynamics. The need for visual signaling and social interactions under the best conditions of the eagle owl daily cycle could represent an alternative, or at least not mutually exclusive, to the proposed [20], [21] but controversial [22] acoustic transmission hypothesis, which predicts that birds are merely taking advantage of better sound propagation close to sunrise and sunset.

In their review of dawn chorusing, Staicer et al. [1] suggested that: “*Many of the hypotheses are weak on explaining timing. A useful hypothesis must show that its proposed function depends on singing before sunrise…This problem can be addressed by collecting data for other species…Light level is obviously an important proximal cue for the time of acoustic signaling in birds, but, surprisingly, no one seems to have addressed why different species are cued by different light intensities*.”

Finally, our explanation for the dawn and dusk chorus of eagle owls may possibly represent an overlooked but common pattern of signaling among crepuscular and nocturnal birds that combine patches of white feathers with twilight displays, such as other owls (e.g. great horned owl *Bubo virginianus*, great grey owl *Strix nebulosa*, little owl *Athene noctua*), great snipes (*Gallinago media* [23], bustards (*Otis tarda*), little bustards (*Tetrax tetrax* [24]) and nightjars [25]. Furthermore, our findings could be relevant to songbirds that breed in dark forest habitats and have contrasting white badges, as well as birds living in open habitats and showing contrasting bars (e.g. *Charadrius* plovers, many larks).

## Materials and Methods

### Field experiments

We conducted experiments in the territories of 25 eagle owl breeding males in the Sierra Norte of Seville (37° 30′ N, 06° 03′ W, SW Spain; details in [26]), between November 2002 and January 2003, i.e. the pre-laying period in our study area.

In order to measure the brightness contrast of the white badge with respect to both the owl body and the background surrounding calling individuals during the period of dusk displays, a stuffed eagle owl was placed in each owl territory and was photographed with its badge exposed as it would be during call displays [13]. This decoy was always positioned to ensure a good listening of the dusk vocal displays of the territorial male. We were careful to avoid placing the mount where it would be directly visible to the bird, as this might interfere with spontaneous calling activity. Moreover, because light conditions could vary on a small scale, the decoy was placed on exposed places under light conditions similar to those of the focal owl [12]. This was possible because the position of calling males was generally predictable, as eagle owls use habitual call posts at sunset, and these were located during prior studies. Because ambient light at sunset and sunrise are similar [9] and eagle owls show both dawn and dusk choruses of similar intensity [12], we considered dusk chorus only. During each of the 25 photographic-listening sessions, we recorded the start and the end of the call activity of each owl to define the temporal range of their dusk choruses.

### Image samples collected during call displays

Based on previous information regarding the vocal behavior of the eagle owl [10], [11], [12], and for each of the 25 calling owls, we took a picture of the decoy every five minutes, from an hour before sunset to 15 minutes after the last call of the dusk display (overall sample size of pictures  = 482). We selected this time bracket because, following previous observations [10], [11], [12]: (a) starting one hour before sunset allows us to have the time to take some pictures before the starting of eagle owl dusk chorus; and (b) generally, after the last call of the dusk chorus owls move to their hunting areas and temporally cease their call activities. The three supplemental pictures that we took during the 15 minutes after the last call allowed us to collect information on brightness after the cease of vocal displays. Pictures were taken with a digital camera Nikon D70 and a 300 mm lens (AF-S Nikkor 1:2.8 G ED) always placed on a tripod at the same distance from the decoy and with the diaphragm set to f.8.

### Measurements of the brightness contrasts

Brightness values of the white badge, the body and the external environment was measured using the Photoshop CS2 software. Using this program, each pixel in an image can be identified across 256 levels of brightness from pure black (0) to pure white (255). A histogram of the intensity of the reflected light shows how all 256 possible levels of brightness are distributed in the image. The horizontal axis of the Photoshop histogram represents the range of brightness from 0 on the left to 255 on the right, i.e. a line with 256 spaces on which to stack pixels of the same brightness. Measurements were performed as follows in the overall sample size of 482 pictures. First, the digital pictures were modified to the grayscale mode and a Gaussian blur filter (radio  = 5.0) was applied to diffuse the image and to avoid alteration of brightness by “outlier” pixels. Next, measurement patches were randomly selected from the white badge, the body and the external environment of each picture. For this purpose we applied a grid of 260 squares (size  = 50×50 pixels) to the photograph. Each square was numbered, squares on the badge-body and body-environment borderlines were excluded (as noise could bias measurements), and the measurement squares were chosen using an aleatory method. The mean brightness values of each square were obtained by superposing a rectangular tool of 50×50 pixels over each measurement patch and using the eyedropper tool; results were reported on the histogram X-axis of the picture as mean±SD. Contrasts between the white badge and the background or body were measured as the differences between the values of mean brightness recorded for each square.

### Statistical analyses

We used Generalized Linear Mixed Models [27] to test whether the contrasts of the white badge *vs*. background and white badge *vs*. body differed: (1) during the pre-calling period, i.e. the period of time before the start of the first owl bout; (2) when owls were calling, i.e. the period of owl call activity; and (3) during the post-calling period, i.e. the period of time after the end of the last owl bout, when owls were silent. The statistical analyses were performed with the SAS macro program, GLIMMIX (version 8.2 [28]), which iterates the MIXED procedure (PROC MIXED in the SAS software). The dependent variable was represented by all the brightness scores calculated as the differences between the mean brightness values of white badge *vs*. background and white badge *vs*. body. Mixed-effects models permit both the fixed (i.e. the above cited three calling periods) and random effects (i.e. male owls) to be fitted in a single analysis. Fixed effects model the mean values of the response variable as a function of covariates, while random effects model any patterns in the residuals around these fixed effects, e.g. those generated by repeated observations on the same individual. Because we had repeated measures of the same individuals and wanted to avoid pseudoreplication problems, we considered individuals as a random effect, while the within-individual effects were defined as a fixed factor. Differences among periods were calculated by the LSMEANS statement in SAS. For all analyses, means are given ±SE and statistical significance was set at P<0.05.

## Supporting Information

Figure S1 The different contrasts among the eagle owls' white throat badges, the surrounding background and owl body during the time brackets of the experiment.
(1.58 MB DOC)

Video S1 Eagle owls showing the white badge during vocal displays. The different contrasts among the eagle owls' white throat badges, the surrounding background and owl body during the time brackets of the experiment.
(120.33 MB MPG)
