# Supplementary material for: The Dusk Chorus from an Owl Perspective: Eagle Owls Vocalize When Their White Throat Badge Contrasts Most
Source: PLoS One. 2009 Apr 8;4(4):e4960. doi: 10.1371/journal.pone.0004960 (PMC2662407; doi:10.1371/journal.pone.0004960)
Supplement: Figure S1 — The different contrasts among the eagle owls' white throat badges, the surrounding background and owl body during the time brackets of the experiment. (1.58 MB DOC) [file pone.0004960.s001.doc]

# Supporting Information 2 (Figure S1)

A visual example of the way the eagle owls’ white throat badges contrasted most with the surrounding background and their body during the dusk chorus (three case studies). A picture for each of the three periods (i.e. the pre-calling, the calling and the post-calling periods) is shown. Box-plots show the brightness contrast during the three periods for the overall data.

**PRE-CALLING PERIOD CALLING PERIOD POST-CALLING PERIOD**


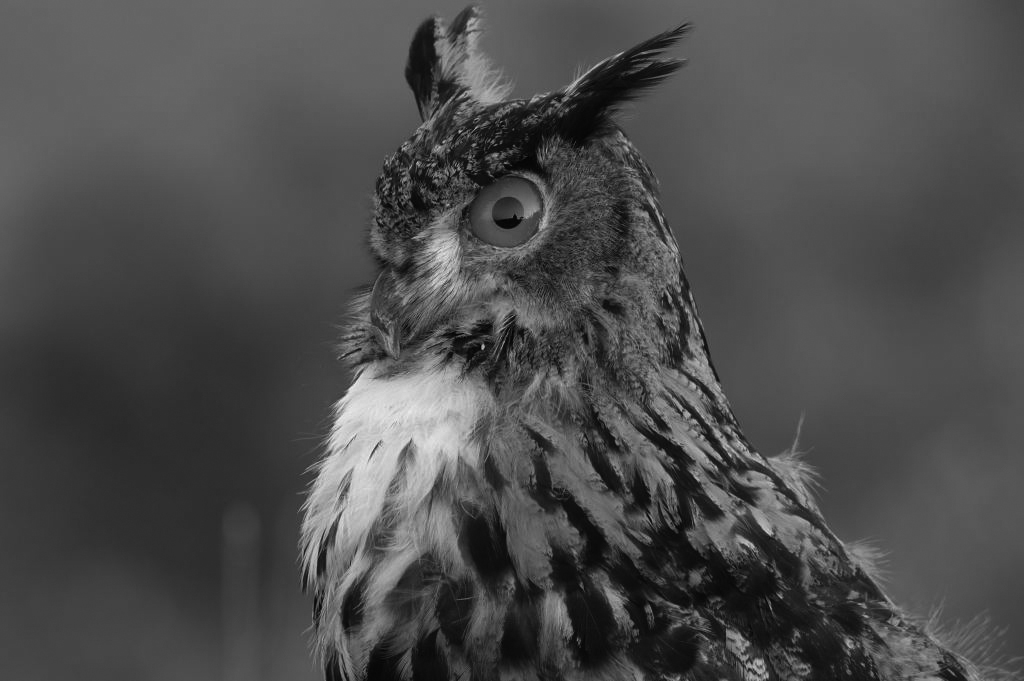

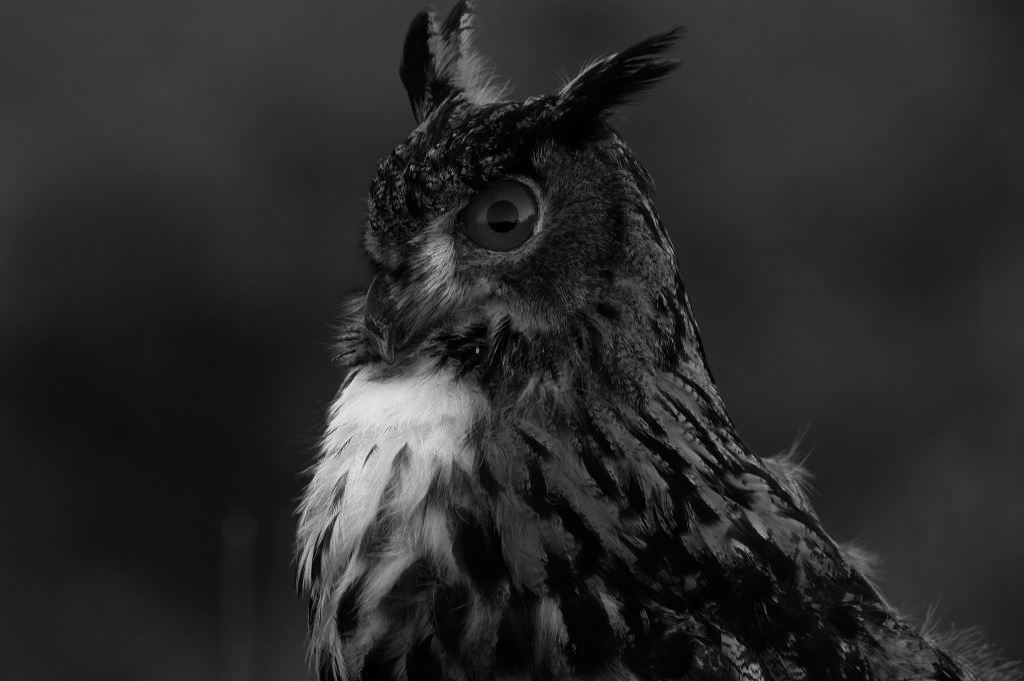

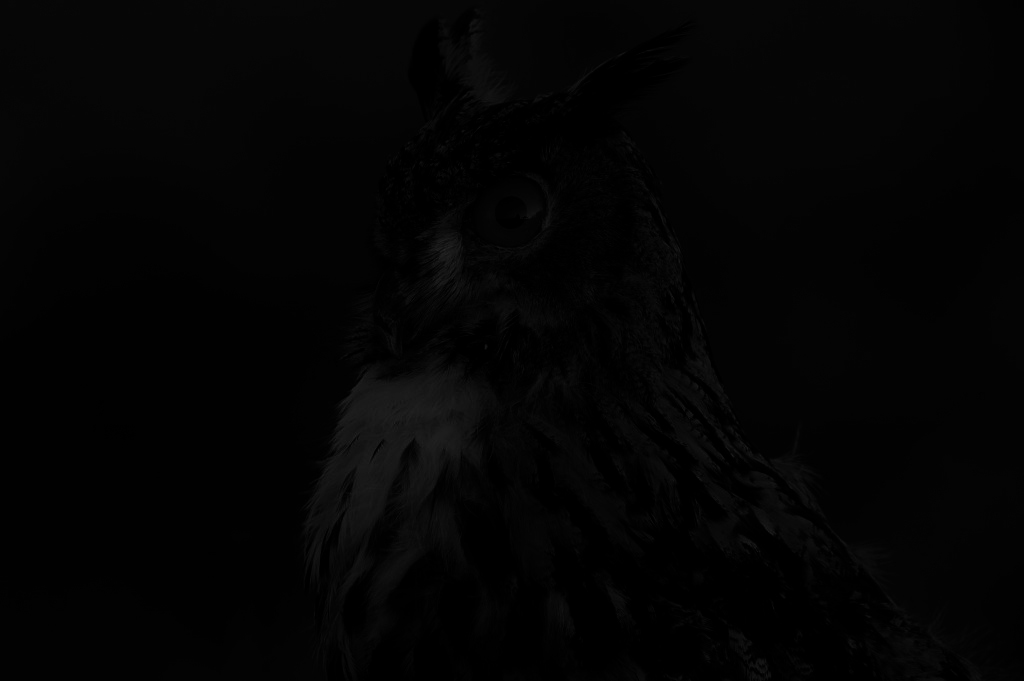


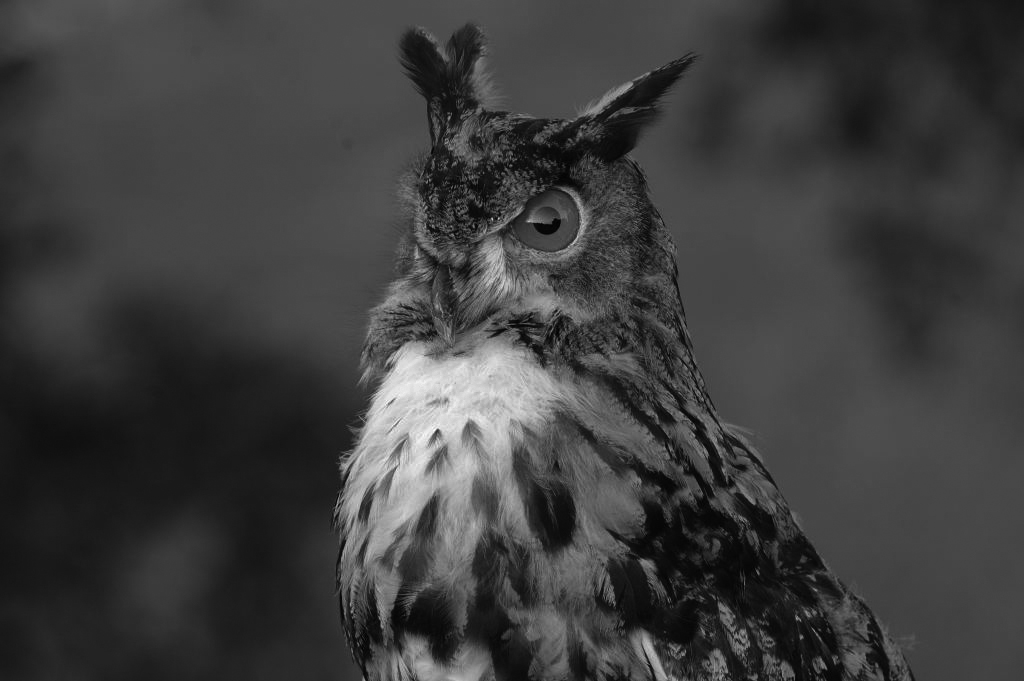

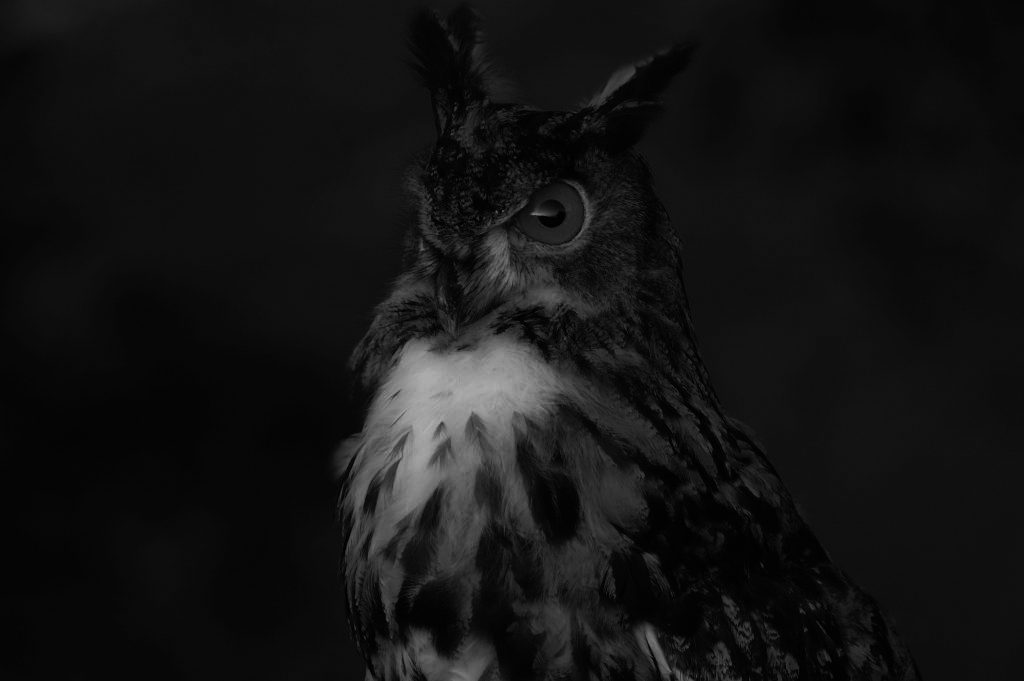

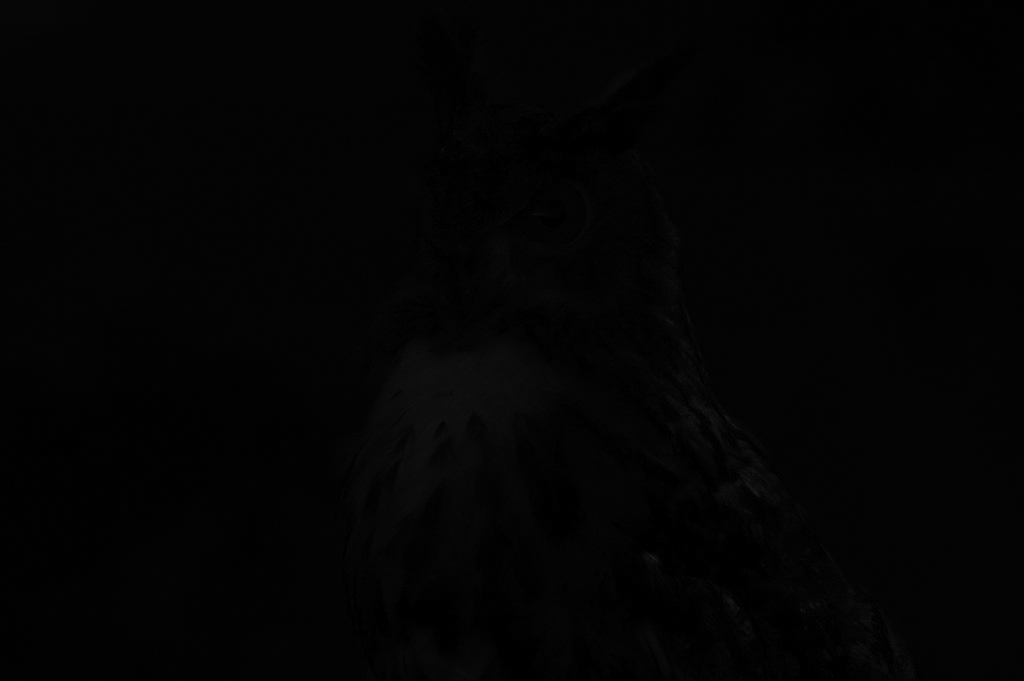


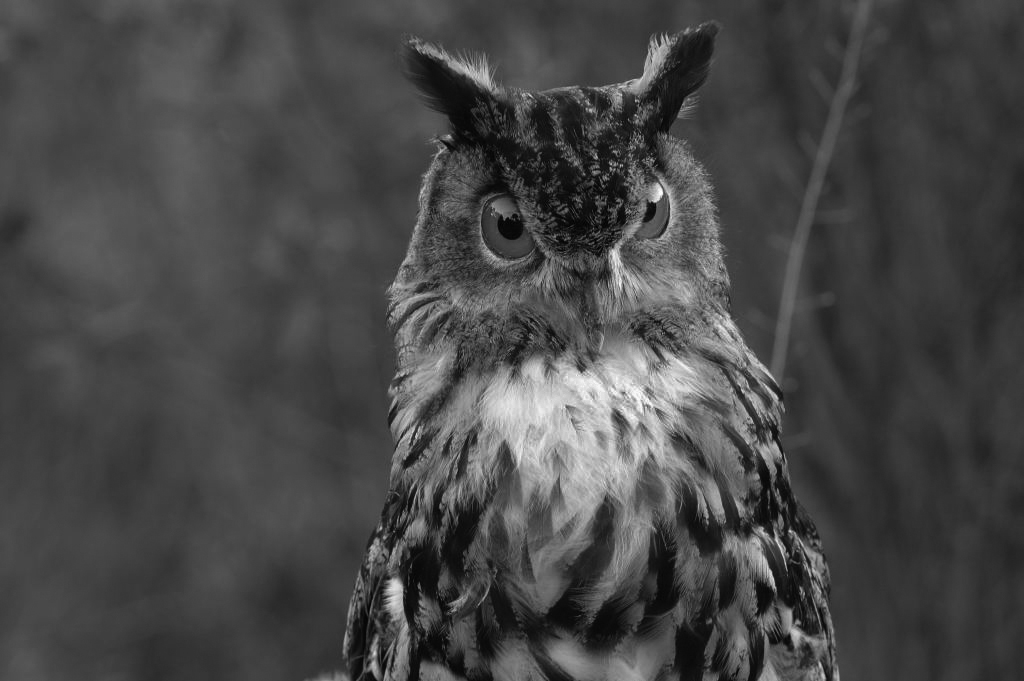

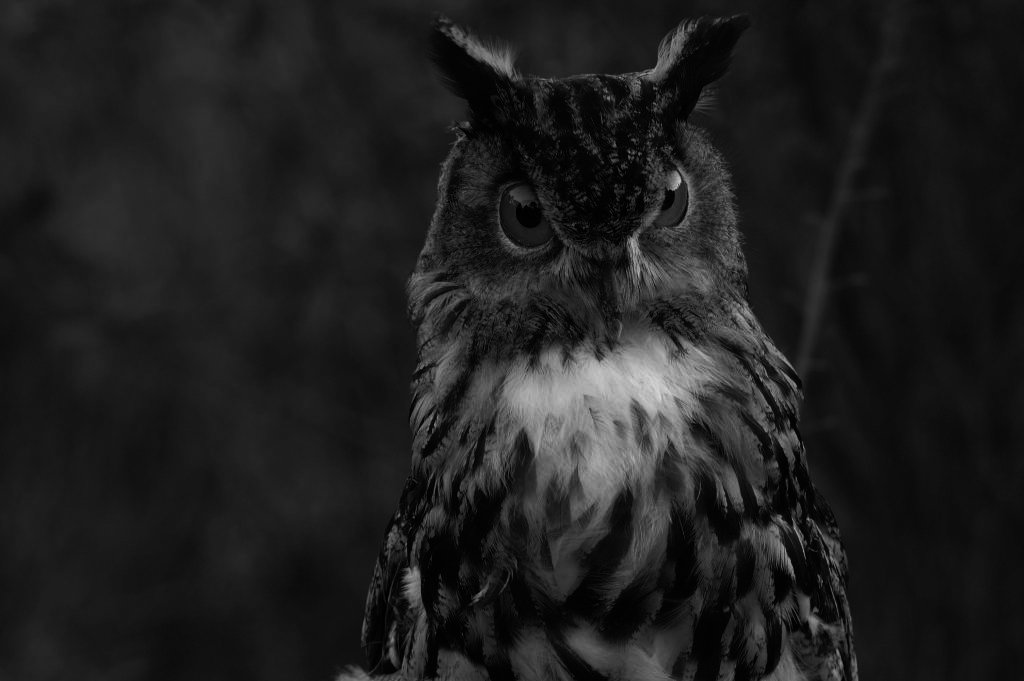

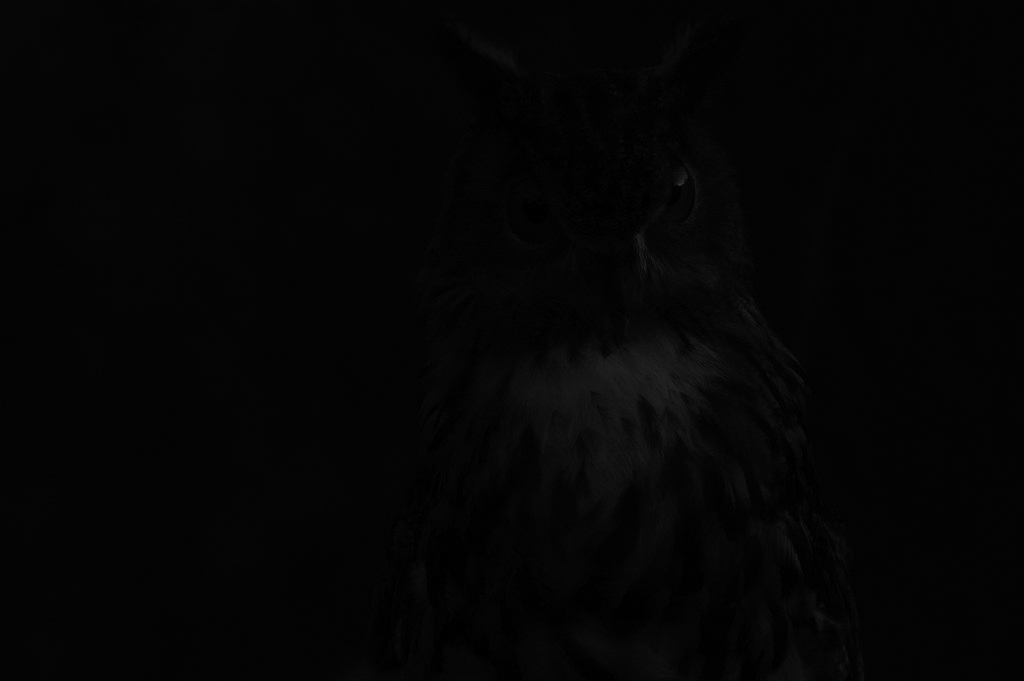


**A** **B**
